# Androgen profiling in adolescent girls with polycystic ovary syndrome

**DOI:** 10.3389/fendo.2026.1817505

**Published:** 2026-06-03

**Authors:** Xuan Zhang, Yanan Zhang, Hemeng Chong, Yutong Xing, Xuehan Gao, Yueqiu Qin, Xujing Kong, Susu Cai, Baorong Chen, Xue Yan, Huifeng Zhang

**Affiliations:** 1 Department of Pediatrics, The Second Hospital of Hebei Medical University, Shijiazhuang, China; 2 Kingmed Diagnostics (Beijing) Co., Ltd, Beijing, China

**Keywords:** adrenal glands, androgens, dexamethasone suppression test, liquid chromatography–mass spectrometry, ovary

## Abstract

**Objective:**

To characterize global steroid hormone dysregulation and the androgen profile in adolescent girls with polycystic ovary syndrome (PCOS), and to explore adrenal versus ovarian androgen origins using a dexamethasone suppression test combined with liquid chromatography-tandem mass spectrometry (LC-MS/MS).

**Methods:**

We consecutively enrolled 37 adolescent PCOS patients and 22 age-matched healthy controls. PCOS diagnosis followed the 2023 international evidence-based recommendations. All participants underwent clinical evaluation and LC-MS/MS steroid profiling; 24 PCOS patients also received a dexamethasone suppression test.

**Results:**

Compared with healthy controls, the PCOS group had elevated levels of multiple androgens, including dehydroepiandrosterone (DHEA), dehydroepiandrosterone sulfate (DHEA-S), androstenedione (AD), total testosterone (TT), androsterone (ADT), dihydrotestosterone (DHT), 11β-hydroxyandrostenedione (11-OHAD), 11β-hydroxytestosterone (11-OHT), and epitestosterone (EpiT), as well as estrone, pregnenolone, and 17-hydroxyprogesterone, along with decreased sex hormone-binding globulin (SHBG). Spearman correlation showed that DHEA-S explained only limited variation in 11-OHAD (r_s_ = 0.629, *P* < 0.001). ROC analysis identified the free androgen index (FAI) as the best diagnostic marker (AUC = 0.976, sensitivity 91.7%, specificity 87.0% at cutoff 4.27). FAI alone was elevated in 86.5% of PCOS patients; adding total testosterone and androstenedione increased detection to 97.3%. In the dexamethasone suppression test, DHEA, DHEA-S, and 11-oxygenated androgens were markedly suppressed (median inhibition 81.9%–92.2%), whereas testosterone suppression was minimal (14.0%, *P* = 0.522). Notably, baseline DHEA-S was not elevated in most patients with significant androgen suppression, indicating discordance between suppression-test-defined adrenal dominance and traditional DHEA-S criteria.

**Conclusion:**

Adolescent girls with PCOS exhibit widespread androgen abnormalities. FAI demonstrates the highest diagnostic value, and the addition of total testosterone and androstenedione modestly improves detection. The dexamethasone suppression test suggests that most 11-oxygenated androgens are of adrenal origin, whereas testosterone suppression is minimal. Elevated DHEA-S levels are not consistently aligned with adrenal-origin androgen dominance defined by the suppression test. These findings warrant further validation in larger populations.

## 1 Introduction

Polycystic ovary syndrome (PCOS) is the most common endocrine disorder in women of reproductive age, affecting 5% to 15% of women depending on the diagnostic criteria used (1). Adolescence is a critical period for PCOS onset, but its diagnosis is challenging due to physiological overlap with normal puberty. According to the 2023 international evidence-based guidelines (2), adolescent PCOS requires the presence of menstrual irregularities combined with clinical and/or biochemical hyperandrogenism, after exclusion of other disorders. Pelvic ultrasound findings and anti-Müllerian hormone levels should not be used for diagnosis in this age group. Adolescents who meet only one criterion (menstrual irregularities or hyperandrogenism) are considered at high risk and require longitudinal follow-up (2, 3).

Clinical hyperandrogenism (e.g., hirsutism, acne) does not always fully correlate with biochemical hyperandrogenemia (elevated serum androgens) (4). Androgens comprise a family of hormones, including testosterone, androstenedione, dehydroepiandrosterone (DHEA), dehydroepiandrosterone sulfate (DHEA-S), and the recently recognized 11-oxygenated androgens (5, 6). Relying solely on a few markers cannot capture the full hyperandrogenemia status. Because adolescent hyperandrogenemia is an early precursor of adult PCOS (2, 3). Its accurate assessment is crucial for early intervention.

The pathogenesis of PCOS involves genetic, metabolic, and environmental interactions. Genome-wide association studies have identified approximately 30 susceptibility loci, with DENND1A confirmed as a core regulator of theca cell androgen synthesis. Most PCOS cases represent a “functional typical” form of ovarian hyperandrogenism, characterized by a hypersensitive steroidogenic response to gonadotropins due to an activated DENND1A variant (7). Obesity-aggravated insulin resistance leads to compensatory hyperinsulinemia, which synergizes with elevated luteinizing hormone to further enhance ovarian androgen production. Approximately one-third of adult PCOS cases exhibit a “functional atypical” form, driven by severe obesity and pro-inflammatory adipokines (7). Thus, intrinsic ovarian defects are the primary cause of hyperandrogenemia, while insulin resistance and obesity play modulatory roles. In a subset of patients, adrenal-derived androgens also contribute significantly. Premature adrenarche, especially when combined with obesity, may serve as a sequential trigger for PCOS through excessive adrenal androgen secretion (8). Hence, hyperandrogenemia in PCOS is heterogeneous, with androgen excess originating from the ovaries, adrenals, or both (9).

This etiological heterogeneity complicates clinical evaluation. Traditional assessment relies on a few hormones (e.g., total testosterone, DHEA-S), which fails to reveal the full androgen profile. Adrenal-derived 11-oxygenated androgens (e.g., 11β-hydroxyandrostenedione, 11-ketotestosterone) are a typical example (10, 11). Additionally, abnormal activation of the “backdoor pathway” to dihydrotestosterone may occur, manifesting as elevated androsterone and dihydrotestosterone (12, 13).

Therefore, to investigate the origin of excess androgens (ovarian vs. adrenal) and to comprehensively profile serum androgens (including classic, 11-oxygenated, and backdoor pathway metabolites) in adolescent girls with PCOS, we performed a dexamethasone suppression test combined with LC-MS/MS. This study combined these two approaches to explore the heterogeneity of hyperandrogenemia in this population.

## 2 Materials and methods

### 2.1 Study participants

This study was approved by the Ethics Committee of The Second Hospital of Hebei Medical University. Written informed consent was obtained from all participants and their parents in accordance with the Declaration of Helsinki. Adolescent girls diagnosed with polycystic ovary syndrome (PCOS) were consecutively enrolled from the Pediatric Endocrinology Clinic of The Second Hospital of Hebei Medical University between June 2021 and March 2026. The diagnosis of PCOS in adolescents followed the 2023 international evidence-based recommendations (2), requiring the simultaneous presence of menstrual irregularities and clinical and/or biochemical hyperandrogenism, after exclusion of other etiologies. Menstrual irregularities were defined according to time since menarche: for girls with menarche 1 to <3 years prior, menstrual cycle <21 days or >45 days; for girls with menarche ≥3 years prior, menstrual cycle <21 days or >35 days, or fewer than 8 menstrual cycles per year; or any single cycle >90 days occurring >1 year after menarche. Hyperandrogenism included both clinical and biochemical manifestations. Clinical hyperandrogenism was defined as moderate-to-severe acne or hirsutism (modified Ferriman-Gallwey score ≥6). This mFG score cutoff is based on the 95th percentile for Chinese girls <2 years post-menarche (14). For biochemical hyperandrogenism, total testosterone and free testosterone were used as first-line indicators, with free testosterone assessed by calculating the free androgen index (FAI). If both total testosterone and free testosterone levels were within normal ranges, androstenedione and dehydroepiandrosterone sulfate (DHEA-S) were further referenced. All steroid hormone measurements were performed using validated tandem mass spectrometry (LC-MS/MS). Biochemical hyperandrogenemia was defined as hormone levels exceeding the 95th percentile of the healthy control group. The 5th–95th percentile reference intervals (covering 90% of the control population) are provided in **Table 1**. Exclusion criteria included confirmed Cushing’s syndrome, congenital adrenal hyperplasia, thyroid disorders, androgen-secreting tumors, hyperprolactinemia, and use of medications affecting endocrine metabolism (e.g., androgens, estrogens/progestins, or their analogs/antagonists). Based on the above criteria, a total of 37 adolescent girls with PCOS were enrolled.

**Table 1:** Comparison of clinical characteristics and hormone levels between adolescents with PCOS and healthy controls.

Parameter	PCOS group (n=37)	Control group (n=22)	5th (Control)	95th (Control)	*P* value	Trend
Clinical characteristics						
Age (years)	13.55 ± 1.76^a^	13.10 ± 0.90^a^	—	—	0.197	
BMI (kg/m²)	28.61 ± 5.94^a^	20.87 ± 2.80^a^	—	—	**<0.001**	P > C
HOMA-IR	4.80 (3.00, 8.17)^b^	3.10 ± 0.90^a^	—	—	**<0.001**	P > C
Hirsutism [n (%)]	22 (59.5)	—	—	—		
Moderate-to-severe acne [n (%)]	21 (56.8)	—	—	—		
Acanthosis nigricans [n (%)]	26 (70.3)	—	—	—		
Reproductive hormones						
LH (mIU/mL)	11.08 (9.07, 15.66)^b^	3.89 ± 1.30^a^	—	—	**< 0.001**	P > C
FSH (mIU/mL)	6.18 ± 1.43^a^	5.80 ± 0.88^a^	—	—	0.274	
AMH (ng/mL)	8.55 ± 4.84^a^	3.53 ± 1.72^a^	—	—	**<0.001**	P > C
INB (pg/mL)	164.36 ± 72.72^a^	95.07 (65.51, 187.62)^b^	—	—	**0.045**	P > C
SHBG (nmol/L)	15.19 (9.94, 20.56)^b^	54.85 ± 20.35^a^	—	—	**< 0.001**	P < C
FAI	14.72 (6.52, 20.80)^b^	1.88 ± 1.33^a^	0.22	4.78	**0.006**	P > C
Androgens						
DHEA (nmol/L; to ng/dL: ×28.84)	13.31 (8.98, 22.78)^b^	5.69 (4.65, 8.53)^b^	2.11	16.57	**0.008**	P > C
DHEA-S (μmol/L; to μg/dL: ×36.85)	4.25 (2.72, 6.25)^b^	2.08 (1.06, 2.75)^b^	0.60	6.31	**0.002**	P > C
AD (nmol/L; to ng/dL: ×28.64)	6.14 (4.28, 9.42)^b^	2.61 ± 1.25^a^	0.46	4.63	**< 0.001**	P > C
TT (nmol/L; to ng/dL: ×28.84)	2.21 ± 0.98^a^	0.85 ± 0.48^a^	0.20	1.72	**0.003**	P > C
ADT (nmol/L; to ng/dL: ×29.04)	1.24 ± 0.49^a^	0.45 ± 0.23^a^	0.09	0.80	**< 0.001**	P > C
DHT (nmol/L; to ng/dL: ×29.04)	0.20 (0.15, 0.27)^b^	0.10 ± 0.06^a^	0.02	0.25	**< 0.001**	P > C
11-OHAD (nmol/L; to ng/dL: ×30.24)	4.46 ± 2.75^a^	2.07 ± 1.03^a^	0.57	4.52	**< 0.001**	P > C
11-KAD (nmol/L; to ng/dL: ×30.04)	0.17 (0.06, 0.38)^b^	0.11 (0.05, 0.19)^b^	0.02	0.52	0.274	
11-KT (nmol/L; to ng/dL: ×30.24)	1.40 ± 0.65^a^	1.00 (0.83, 1.50)^b^	0.48	2.66	0.295	
11-OHT (nmol/L; to ng/dL: ×30.44)	0.25 (0.12, 0.34)^b^	0.09 (0.05, 0.12)^b^	0.02	0.29	**0.003**	P > C
EpiA (nmol/L; to ng/dL: ×29.04)	0.10 (0.06, 0.22)^b^	0.04 (0.02, 0.08)^b^	0.005	0.19	0.164	
EpiT (nmol/L; to ng/dL: ×28.84)	0.12 (0.07, 0.19)^b^	0.03 (0.01, 0.06)^b^	0.002	0.16	**0.002**	P > C
Glucocorticoids						
11DOF (nmol/L; to ng/dL: ×34.65)	1.03 (0.52, 1.85)^b^	0.72 (0.49, 1.19)^b^	—	—	0.183	
F (nmol/L; to ng/dL: ×36.25)	234.23 (143.88, 349.83)^b^	164.15 (106.16, 215.74)^b^	—	—	0.063	
E (nmol/L; to ng/dL: ×36.04)	54.10 (39.53, 64.78)^b^	50.22 (33.43, 63.12)^b^	—	—	0.644	
21DOF (nmol/L; to ng/dL: ×34.65)	0.04 (0.01, 0.07)^b^	0.02 (0.01, 0.03)^b^	—	—	0.353	
18-OHF (nmol/L; to ng/dL: ×37.85)	1.31 (0.84, 1.93)^b^	0.95 (0.44, 1.35)^b^	—	—	0.068	
18-OF (nmol/L; to ng/dL: ×37.64)	0.04 (0.02, 0.06)^b^	0.02 (0.01, 0.05)^b^	—	—	0.132	
Mineralocorticoids						
11-DOC (nmol/L; to ng/dL: ×33.05)	0.08 (0.05, 0.14)^b^	0.10 (0.06, 0.15)^b^	—	—	0.666	
B (nmol/L; to ng/dL: ×34.65)	6.26 (2.80, 14.34)^b^	3.43 (1.96, 5.43)^b^	—	—	0.305	
18-OHB (nmol/L; to ng/dL: ×36.25)	1.24 (0.88, 2.53)^b^	1.57 (0.95, 2.69)^b^	—	—	0.344	
ALD (nmol/L; to ng/dL: ×36.04)	0.13 (0.08, 0.32)^b^	0.25 (0.09, 0.47)^b^	—	—	0.199	
Estrogens						
E1 (pmol/L; to pg/mL: ×0.27)	129.08 (103.38, 177.17)^b^	65.84 (44.75, 97.02)^b^	—	—	**0.023**	P > C
E2 (pmol/L; to pg/mL: ×0.27)	131.98 (84.07, 177.51)^b^	109.41 (35.61, 245.79)^b^	—	—	0.320	
E3 (pmol/L; to pg/mL: ×0.29)	2.29 (1.46, 6.17)^b^	2.88 (1.49, 6.55)^b^	—	—	0.754	
Progestogens						
PREG (nmol/L; to ng/dL: ×31.65)	1.11 (0.55, 1.60)^b^	0.68 ± 0.34^a^	—	—	**0.027**	P > C
P (nmol/L; to ng/dL: ×31.45)	0.22 (0.16, 0.32)^b^	0.22 (0.13, 1.78)^b^	—	—	0.906	
17-OHPREG (nmol/L; to ng/dL: ×33.25)	4.39 (2.92, 6.98)^b^	2.26 (1.44, 3.49)^b^	—	—	0.194	
17-OHP (nmol/L; to ng/dL: ×33.05)	2.06 (1.39, 2.78)^b^	1.15 (0.82, 1.76)^b^	—	—	**0.036**	P > C

^a^ Data are mean ± SD;

^b^ Data are median (25th–75th percentile).

Group comparisons were performed using independent samples t-test or Mann-Whitney U test accordingly. *P* < 0.05 was considered statistically significant. “—” indicates no data/not measured, or that the 5th and 95th percentiles are not shown (reference ranges are provided only for androgens and FAI). Hirsutism was defined as a modified Ferriman-Gallwey score of ≥6. P > C indicates higher values in the PCOS group than in the control group; P < C indicates lower values in the PCOS group. To account for the significant BMI difference between groups, all between-group comparisons of steroid hormones (including androgens, glucocorticoids, mineralocorticoids, estrogens, and progestogens) and the FAI were additionally analyzed using linear regression with bootstrapping (1000 resamples) and BMI as a covariate. The *P* values presented for these steroid hormones and FAI are those after BMI adjustment. *P*-values lower than 0.05 are marked in bold. In the control group, the 5th and 95th percentiles are presented for 12 androgens and the FAI to establish reference ranges; all these percentiles are empirical values calculated directly from the raw data. Conversion factors from SI units to conventional units (ng/dL for most steroids, μg/dL for DHEA-S, pg/mL for estrogens) are provided in parentheses next to each steroid in the Parameter column. For example, for DHEA (nmol/L), multiply by 28.84 to obtain ng/dL. BMI, body mass index; HOMA-IR, homeostatic model assessment of insulin resistance; LH, luteinizing hormone; FSH, follicle-stimulating hormone; AMH, anti-Müllerian hormone; INB, inhibin B; SHBG, sex hormone-binding globulin; FAI, free androgen index; DHEA, dehydroepiandrosterone; DHEA-S, dehydroepiandrosterone sulfate; AD, androstenedione; TT, total testosterone; ADT, androsterone; DHT, dihydrotestosterone; 11-OHAD, 11β-hydroxyandrostenedione; 11-KAD, 11-ketoandrostenedione; 11-KT, 11-ketotestosterone; 11-OHT, 11β-hydroxytestosterone; EpiA, epiandrosterone; EpiT, epitestosterone. 11DOF, 11-deoxycortisol; F, cortisol; E, cortisone; 21DOF, 21-deoxycortisol; 18-OHF, 18-hydroxycortisol; 18-OF, 18-oxocortisol; 11-DOC, 11-deoxycorticosterone; B, corticosterone; 18-OHB, 18-hydroxycorticosterone; ALD, aldosterone; E1, estrone; E2, estradiol; E3, estriol; PREG, pregnenolone; P, progesterone; 17-OHPREG, 17-hydroxypregnenolone; 17-OHP, 17-hydroxyprogesterone. *P*-values lower than 0.05 are marked in bold.

During the same period, age-matched healthy girls were recruited as controls from the same clinic, with a total of 22 participants enrolled. Control group inclusion criteria were: menarche >1 year prior, regular menstrual cycles (within normal range), no clinical manifestations of hyperandrogenism (moderate-to-severe acne, hirsutism, or menstrual irregularities), no biochemical hyperandrogenism, and no obesity. All control participants were also screened to exclude Cushing’s syndrome, congenital adrenal hyperplasia, thyroid disorders, androgen-secreting tumors, and hyperprolactinemia, and were not taking medications affecting endocrine metabolism.

### 2.2 Clinical assessment and sample collection

All participants underwent systematic physical examinations and clinical data collection by professional physicians. Age, height, and weight were recorded, and body mass index (BMI) was calculated accordingly. Pubertal development was assessed using Tanner staging, and the presence of moderate-to-severe acne and acanthosis nigricans was documented. Hirsutism was evaluated using the modified Ferriman-Gallwey score, with a score of ≥4–6 defined as hirsutism. After completing the above assessments, peripheral venous blood was collected in the morning under fasting conditions at 8:00 AM. Serum samples were used to measure reproductive hormones, including luteinizing hormone, follicle-stimulating hormone, anti-Müllerian hormone, inhibin B, and sex hormone-binding globulin. Plasma samples were used to quantify 29 steroid hormones by liquid chromatography-tandem mass spectrometry (LC-MS/MS). The homeostatic model assessment of insulin resistance (HOMA-IR) was calculated based on fasting glucose and insulin levels. The free androgen index (FAI) was calculated as [total testosterone (nmol/L)/SHBG (nmol/L)] × 100.

### 2.3 Dexamethasone suppression test

To identify the origin of hyperandrogenemia, 24 PCOS patients underwent low-dose and high-dose dexamethasone suppression tests. The test was initiated at 8:00 AM on the first day. Patients received oral dexamethasone 0.5 mg every 6 hours for 2 consecutive days (low-dose regimen), followed by oral dexamethasone 2 mg every 6 hours for 2 consecutive days (high-dose regimen). Blood samples were collected at baseline (before medication), after low-dose suppression, and after high-dose suppression. Plasma levels of glucocorticoids, mineralocorticoids, androgens, estrogens, and progestogens were measured by liquid chromatography-tandem mass spectrometry (LC-MS/MS). Referring to the method described by Chen et al. (15), a decrease of more than 50% in serum testosterone and androstenedione levels from baseline after dexamethasone suppression was defined as the criterion for significant suppression. If the hormone level decreased to less than 50% of the baseline value, the hormone was considered to be primarily of adrenal origin; otherwise, it was considered to be primarily of ovarian origin.

### 2.4 Routine biochemical and immunoassay measurements

Fasting glucose, fasting insulin, luteinizing hormone (LH), follicle-stimulating hormone (FSH), anti-Müllerian hormone (AMH), inhibin B (INB), and sex hormone-binding globulin (SHBG) were all measured in the Department of Clinical Laboratory at The Second Hospital of Hebei Medical University. This laboratory is accredited by the China National Accreditation Service for Conformity Assessment (CNAS) under ISO 15189. Fasting glucose was measured using the glucose oxidase method. Fasting insulin, LH, FSH, AMH, INB, and SHBG were measured by electrochemiluminescence immunoassay using a Roche E170 electrochemiluminescence analyzer (Roche Diagnostics, Germany). All measurements were performed strictly in accordance with the instrument and kit instructions. Each batch of testing included quality control materials, and test reports were issued only after quality control specifications were met.

### 2.5 Steroid hormone profiling

Steroid hormones were measured by liquid chromatography-tandem mass spectrometry (LC-MS/MS) using a laboratory-developed test (LDT) (16) established in strict accordance with the CLSI C62 guideline, second edition. Our method mainly uses a C8−packed column with gradient elution to achieve separation and simultaneous measurement of different classes of steroid hormones. For example, Braun et al. successfully separated DHEA−S from DHEA and other steroids using a similar LC−MS/MS setup (17). The instrumentation consisted of a Waters UPLC-MS/MS system (model: ACQUITY I-Class/Xevo TQ-S; Waters Corporation, USA) equipped with a Waters BEH C8+ column (2.1 mm × 100 mm, 1.7 μm). The column temperature was maintained at 50 °C, and the autosampler temperature was set at 8 °C. The mobile phase consisted of 0.5 mM ammonium fluoride in water (A) and methanol (B), with flow rates of 0.3 mL/min for mobile phase A and 0.5 mL/min for mobile phase B. The elution gradient is provided in **Supplementary Table 7**. Mass spectrometry was performed using the Xevo TQ-S MS system with optimized parameters as follows: capillary voltage 2.80 kV, ion source temperature 150 °C, desolvation temperature 500 °C, cone gas flow 150 L/h, desolvation gas flow 1100 L/h, and collision gas flow 0.15 mL/min. The mass spectrometric acquisition parameters for each analyte are provided in **Supplementary Table 8**. Methodological performance characteristics (precision, recovery, linear range, limit of detection, and limit of quantification) for each analyte are detailed in **Supplementary Table 6**. Information on chemical reagents, standards, and internal standards is provided in **Supplementary Table 9**. The Beijing KingMed Diagnostics Laboratory is accredited by the China National Accreditation Service for Conformity Assessment (CNAS) under ISO 15189 (certificate number: MT0613) (www.cnas.org.cn). At least two concentration levels of internal quality controls were included in each batch, and clinical sample testing was performed only after quality control specifications were met. The laboratory participates annually in the external quality assessment program of the National Center for Clinical Laboratories. During the study period, all test items passed the external quality assessment criteria (laboratory code: 101801) (http://www.nccl.org.cn). In addition, progesterone, testosterone, estradiol, 17-hydroxyprogesterone, aldosterone, cortisol, and estriol participated in the IFCC-RfB-RELA inter-laboratory comparison program, and all quality control results met the required standards (laboratory code: 152) (https://www.dgkl-rfb.de-RELA Homepage).

The panel of steroid hormones measured included: dehydroepiandrosterone, dehydroepiandrosterone sulfate, androstenedione, testosterone, dihydrotestosterone, androsterone, epiandrosterone, epitestosterone, 11β-hydroxyandrostenedione, 11-ketoandrostenedione, 11β-hydroxytestosterone, 11-ketotestosterone, pregnenolone, progesterone, 17-hydroxypregnenolone, 17-hydroxyprogesterone, estrone, estradiol, estriol, cortisol, cortisone, 11-deoxycortisol, 21-deoxycortisol, 18-hydroxycortisol, 18-oxocortisol, 11-deoxycorticosterone, corticosterone, 18-hydroxycorticosterone, and aldosterone. For values below the lower limit of quantification (LOQ), LOQ/2 was used for statistical analysis.

### 2.6 Statistical analysis

Data were first tested for normality using the Shapiro-Wilk method. Continuous variables with a normal distribution were expressed as mean ± standard deviation (x̄ ± s), while those with a non-normal distribution were expressed as median (25th percentile, 75th percentile). For comparisons between two groups, the independent samples t-test (for normally distributed data) or the Mann-Whitney U test (for non-normally distributed data) was used. To account for the BMI difference between groups, all between-group comparisons of steroid hormones and the free androgen index (FAI) were additionally analyzed using linear regression with bootstrapping (1000 resamples) and BMI as a covariate. This method does not assume normality. For data with three repeated measurements, if the data at all three time points followed a normal distribution, repeated-measures analysis of variance was used for overall comparison. If Mauchly’s test of sphericity was significant (*P* < 0.05), the Greenhouse-Geisser method was applied to correct the degrees of freedom, and *post-hoc* pairwise comparisons were performed using the paired t-test with Bonferroni correction. If the data at any time point did not follow a normal distribution, the Friedman test was used for overall comparison, and *post-hoc* pairwise comparisons were performed using the Wilcoxon signed-rank test with Bonferroni correction. All *P* values reported in the tables are Bonferroni-corrected, and a *P* value < 0.05 was considered statistically significant. Spearman’s rank correlation test was used to assess bivariate associations. To compare correlation coefficients between independent groups (PCOS vs controls), Fisher’s Z transformation was applied. To compare dependent correlations (i.e., DHEA vs DHEA-S with the same 11-oxygenated androgen), Steiger’s Z test was used. All calculations were performed using an online calculator (https://www.psychometrica.de/correlation.html). For independent correlations, the calculation followed Eid et al. (2017, p. 577; single-sided test); for dependent correlations, the calculation followed Eid et al. (2017, p. 578 f.; single-sided test) (18). All statistical analyses were completed using SPSS version 26.0.

## 3 Results

### 3.1 Comparison of clinical characteristics and steroid hormone levels

This study included 37 adolescent PCOS patients and 22 age-matched healthy controls. **Table 1** shows the comparison of clinical characteristics and hormone levels between the two groups. In terms of clinical manifestations, the incidence rates of hirsutism, acne, and acanthosis nigricans in the PCOS group were 59.5%, 56.8%, and 70.3%, respectively. For reproductive hormones, the PCOS group had significantly higher levels of LH, AMH, INB, and FAI than the control group (all *P* < 0.05). In contrast, SHBG levels were significantly lower in the PCOS group (*P* < 0.001). No significant difference was found in FSH levels between the two groups (*P* = 0.274). For the androgen profile, the PCOS group showed significantly higher levels of DHEA, DHEA-S, AD, TT, ADT, DHT, 11-OHAD, 11-OHT, and EpiT than the control group (all *P* < 0.05). However, no significant differences were observed in 11KAD or 11KT levels between the two groups (both *P* > 0.05).

For estrogens and progestogens, the PCOS group had significantly higher levels of estrone, pregnenolone, and 17−hydroxyprogesterone than the control group (all P < 0.05). Regarding glucocorticoids and mineralocorticoids, cortisol, 21−deoxycortisol, and corticosterone were significantly elevated in the PCOS group before BMI adjustment (all P < 0.05; these unadjusted P values are not presented in **Table 1**, which only shows the P values after BMI adjustment). However, after adjusting for BMI using linear regression with bootstrapping (1000 resamples), these differences became non−significant (all P > 0.05), indicating that the elevations of these three hormones were largely influenced by BMI. No other glucocorticoids or mineralocorticoids showed significant differences between groups either before or after BMI adjustment (all P > 0.05).

### 3.2 Spearman correlations of androgen markers in adolescent PCOS girls and healthy controls

Spearman rank correlation analysis showed multiple significant correlations among androgen markers in the PCOS group (n = 37) and the healthy control group (n = 22) (**Table 2**; **Supplementary Table 1**).

**Table 2:** Spearman correlation matrix of androgen parameters in the PCOS group.

Variable	DHEA	DHEA-S	AD	TT	ADT	DHT	11-OHAD	11-KAD	11-KT	11-OHT	EpiA	EpiT
DHEA	1.000	0.771***	0.481**	-0.241	0.425**	0.056	0.782***	0.570***	0.510**	0.569***	0.250	-0.064
DHEA-S	0.771***	1.000	0.202	-0.381*	0.280	-0.100	0.629***	0.462**	0.362*	0.483**	0.285	-0.170
AD	0.481**	0.202	1.000	0.458**	0.461**	0.363*	0.630***	0.299	0.469**	0.500**	0.301	0.406*
TT	-0.241	-0.381*	0.458**	1.000	0.003	0.422*	-0.021	-0.318	0.102	0.194	0.150	0.671***
ADT	0.425**	0.280	0.461**	0.003	1.000	0.334*	0.267	0.338*	0.208	0.298	0.202	0.164
DHT	0.056	-0.100	0.363*	0.422*	0.334*	1.000	0.064	-0.002	0.264	0.265	0.224	0.374*
11-OHAD	0.782***	0.629***	0.630***	-0.021	0.267	0.064	1.000	0.610***	0.770***	0.811***	0.173	0.063
11-KAD	0.570***	0.462**	0.299	-0.318	0.338*	-0.002	0.610***	1.000	0.622***	0.473**	0.134	-0.016
11-KT	0.510**	0.362*	0.469**	0.102	0.208	0.264	0.770***	0.622***	1.000	0.723***	0.135	0.119
11-OHT	0.569***	0.483**	0.500**	0.194	0.298	0.265	0.811***	0.473**	0.723***	1.000	0.266	0.067
EpiA	0.250	0.285	0.301	0.150	0.202	0.224	0.173	0.134	0.135	0.266	1.000	0.261
EpiT	-0.064	-0.170	0.406*	0.671***	0.164	0.374*	0.063	-0.016	0.119	0.067	0.261	1.000

Data are presented as Spearman correlation coefficients (r_s_). *P < 0.05, **P < 0.01, ***P < 0.001. N = 37.

In the PCOS group, the adrenal-derived androgens DHEA and DHEA-S were significantly and positively correlated with all 11-oxygenated androgens. The strongest correlation was between DHEA and 11-OHAD (r = 0.782, *P* < 0.001). For classic androgens, AD was positively correlated with TT, ADT, and DHT (r = 0.363–0.461). TT was also positively correlated with DHT (r = 0.422, *P* = 0.010), but was negatively correlated with DHEA-S (r = -0.381, *P* = 0.022). Strong correlations were observed among 11-oxygenated androgens. In addition, EpiT was strongly and positively correlated with TT (r = 0.671, *P* < 0.001), while EpiA showed no significant correlation with any androgen.

In the healthy control group, the correlation patterns showed both similarities and differences compared to the PCOS group. AD was strongly correlated with TT, ADT, and DHT (r = 0.686–0.814, all *P* < 0.001). TT was also strongly and positively correlated with ADT and DHT (r = 0.799 and 0.844, respectively, both *P* < 0.001). Strong correlations were still present among 11-oxygenated androgens. EpiT was positively correlated with AD, TT, ADT, and DHT (r = 0.633–0.712, all *P* < 0.01). Of note, EpiA was negatively correlated with 11-KAD (r = -0.479, *P* = 0.024), a phenomenon not observed in the PCOS group.

**Figure 1:**
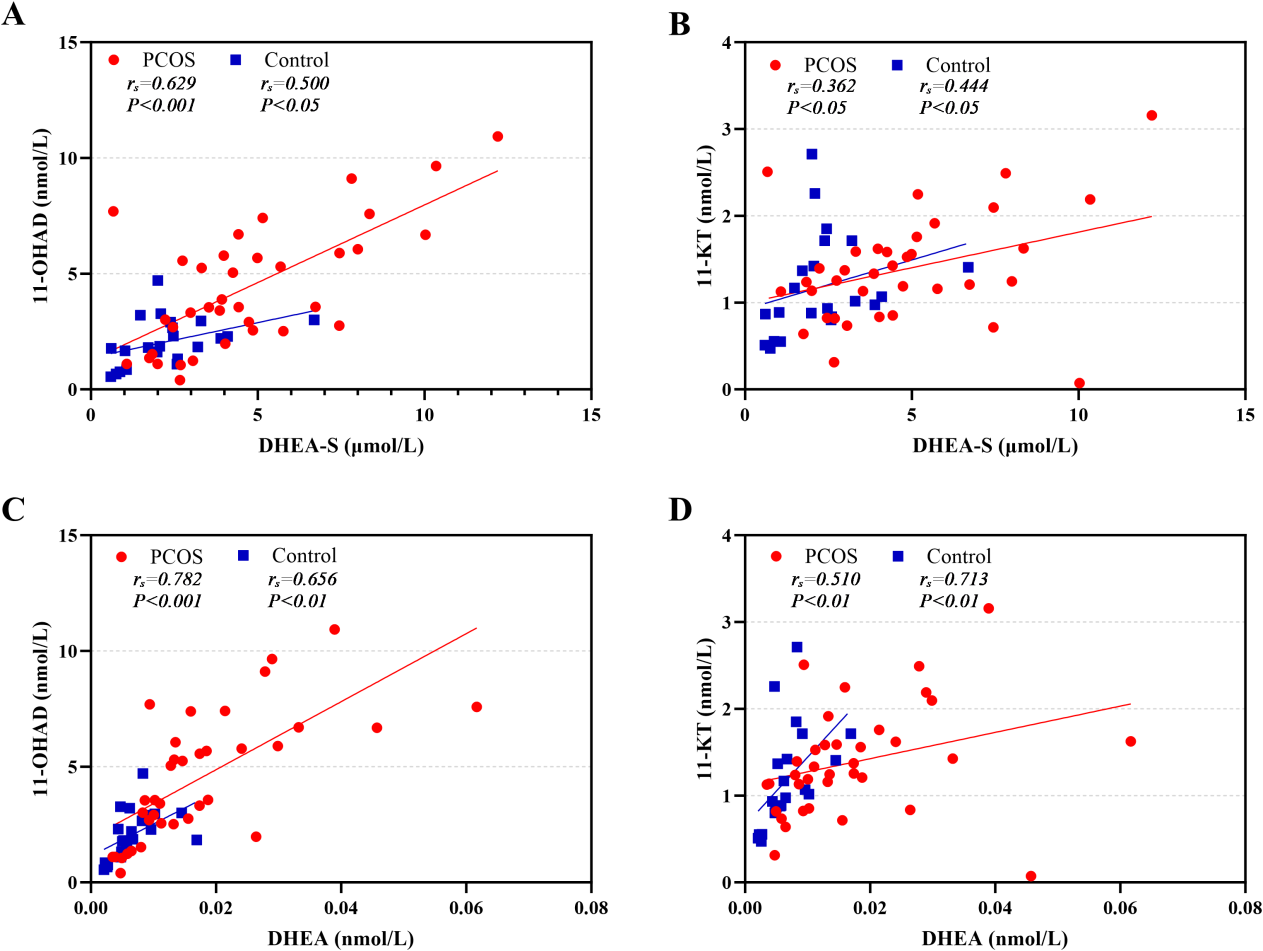
Representative scatter plots of key classic and 11−oxygenated androgens in PCOS patients and healthy controls. Scatter plots of DHEA and DHEA-S versus 11β-hydroxyandrostenedione (11-OHAD) and 11-ketotestosterone (11-KT) in PCOS patients (red circles, n=37) and healthy controls (blue squares, n=22) **(Panels A–D)**. Spearman correlation coefficients (r_s_) and *P*-values are shown on each graph. Linear regression lines are overlaid for visual trend only; statistical inference was based on Spearman’s rank correlation.

To visualize the relationships between classic adrenal androgens and bioactive 11-oxygenated androgens, scatter plots of DHEA and DHEA-S versus 11-OHAD and 11-KT were constructed for both groups (**Figure 1, panels A–D**). In PCOS patients, DHEA-S showed a significant positive correlation with 11-OHAD (panel A: r_s_ = 0.629, *P* < 0.001). The scatter around the regression line suggests that DHEA-S levels account for only about one-third of the variation in 11-OHAD (r_s_ = 0.629, r^2^ ≈ 0.40), indicating that circulating DHEA-S is a weak predictor of this bioactive adrenal androgen precursor.

We further compared the correlation coefficients of adrenal androgens (DHEA and DHEA−S) with 11−oxygenated androgens (11−OHAD and 11−KT) between PCOS patients (n=37) and control subjects (n=22) using Fisher’s Z transformation. As shown in **Supplementary Table 2**, none of the correlations differed significantly between the two groups (all P > 0.05), indicating that for the four correlation pairs examined (DHEA-S and DHEA with 11-OHAD and 11-KT), the relationship patterns did not differ significantly between PCOS and control adolescents.

Within each group, we compared the correlations of DHEA-S versus DHEA with the same 11-oxygenated androgen using Steiger’s Z test. The results are summarized in **Supplementary Table 3**. In PCOS patients, DHEA correlated more strongly with 11-OHAD than did DHEA-S (r = 0.782 vs 0.629, P = 0.022), whereas no significant difference was observed for 11-KT (P = 0.075). In control subjects, DHEA correlated more strongly with 11-KT than did DHEA-S (r = 0.713 vs 0.444, P = 0.031), while the difference for 11-OHAD was not significant (P = 0.144).

### 3.3 Diagnostic value of individual and combined androgen markers

**Figure 2:**
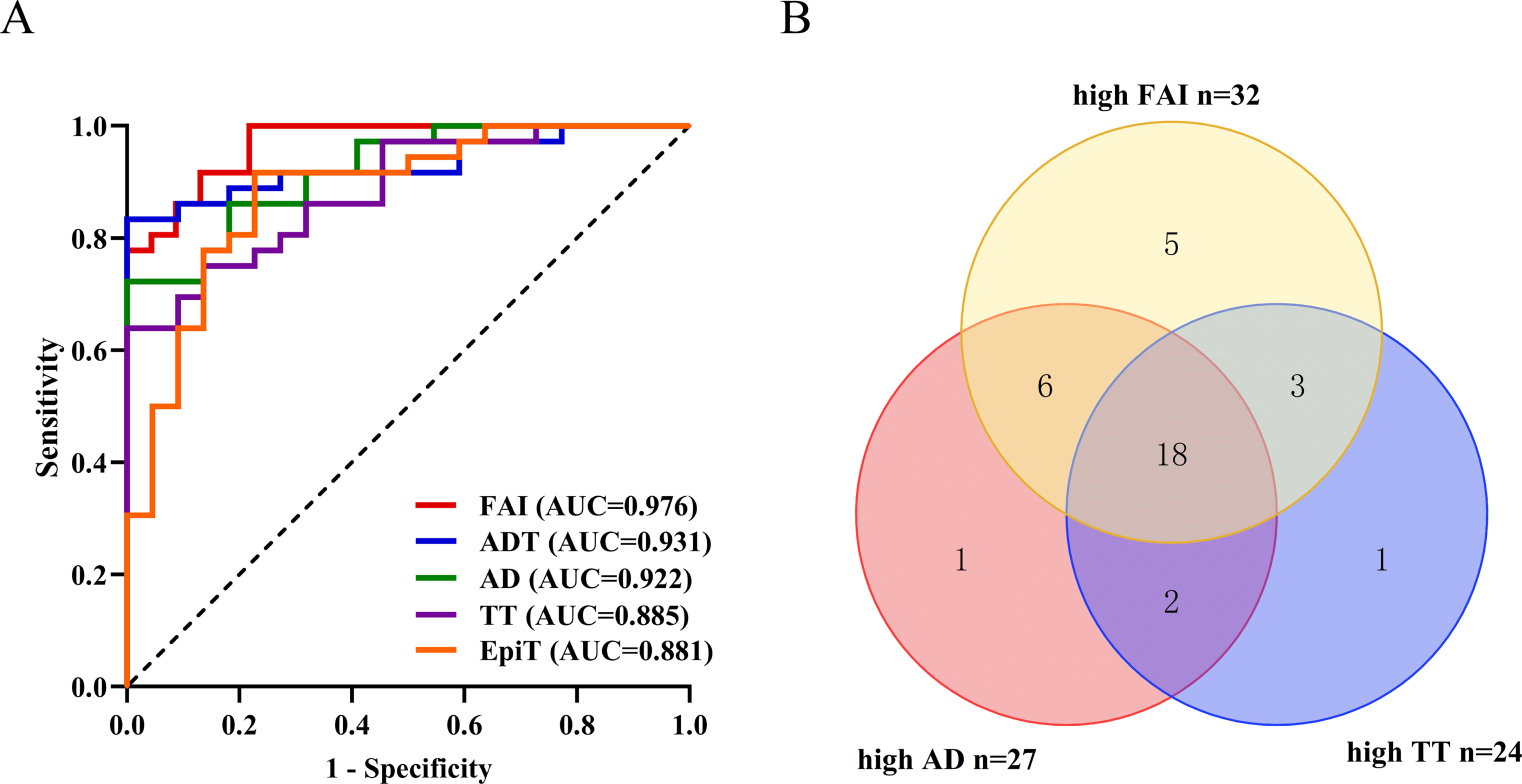
Diagnostic performance of androgens for adolescent PCOS. **(A)** Receiver operating characteristic (ROC) curves of the top five androgen markers (FAI, ADT, AD, TT, and EpiT) for discriminating adolescents with PCOS from healthy controls. FAI showed the highest area under the curve (AUC). **(B)** Venn diagram depicting the diagnostic overlap of FAI, TT, and AD in adolescent PCOS. Using the 95th percentile of healthy controls as thresholds, FAI was elevated in 32/37 patients (86.5%). Adding TT and AD identified an additional four patients (10.8%), resulting in a combined detection rate of 97.3% (36/37). Only one patient (2.7%) with isolated DHEA-S elevation was missed by all three markers. ADT and EpiT added no additional diagnostic value.

To evaluate the diagnostic value of each androgen marker in distinguishing adolescent PCOS patients from healthy controls, we performed ROC curve analysis. **Figure 2A** shows the ROC curves for the top five androgen markers ranked by AUC (FAI, ADT, AD, TT, and EpiT). FAI exhibited the highest diagnostic performance (AUC = 0.976, sensitivity = 91.7%, specificity = 87.0% at the optimal cutoff of 4.27). The other four markers also demonstrated good diagnostic value (AUC range 0.881–0.931). Detailed ROC parameters for all androgens are presented in **Table 3**. Of note, 11KAD and 11KT did not reach statistical significance (*P* > 0.05).

**Table 3:** ROC curve analysis results for all androgen markers in the diagnosis of adolescent PCOS.

Androgens(unit)	AUC	95% CI	*P* value	Optimal cutoff value	Sensitivity (95% CI)	Specificity (95% CI)
FAI	0.976	0.945-1.000	< 0.001	4.27	91.7 (78.0-97.1)	87.0 (68.0-95.5)
ADT (nmol/L)	0.931	0.865-0.996	< 0.001	0.79	83.3 (68.0-92.0)	95.5 (78.0-99.0)
AD (nmol/L)	0.922	0.857-0.986	< 0.001	3.67	86.1 (71.0-94.0)	81.8 (61.0-93.0)
TT (nmol/L)	0.885	0.803-0.967	< 0.001	1.39	75.0 (59.0-86.0)	86.4 (67.0-95.0)
EpiT (nmol/L)	0.881	0.790-0.973	< 0.001	0.05	91.7 (78.0-97.0)	77.3 (57.0-90.0)
DHT (nmol/L)	0.864	0.764-0.964	< 0.001	0.15	80.6 (65.0-90.0)	86.4 (67.0-95.0)
DHEA (nmol/L)	0.835	0.731-0.938	< 0.001	9.22	75.0 (59.0-86.0)	81.8 (61.0-93.0)
11-OHT (nmol/L)	0.826	0.719-0.932	< 0.001	0.15	75.0 (59.0-86.0)	86.4 (67.0-95.0)
DHEA-S (μmol/L)	0.819	0.710-0.929	< 0.001	2.63	80.6 (65.0-90.0)	77.3 (57.0-90.0)
11-OHAD (nmol/L)	0.783	0.666-0.900	< 0.001	3.32	61.1 (45.0-75.0)	95.5 (78.0-99.0)
EpiA (nmol/L)	0.763	0.639-0.886	0.001	0.08	63.9 (48.0-78.0)	86.4 (67.0-95.0)
11-KAD (nmol/L)	0.614	0.468-0.759	0.149	—	—	—
11-KT (nmol/L)	0.607	0.454-0.761	0.173	—	—	—

Data are presented as area under the curve (AUC) with 95% confidence intervals (CI). Optimal cutoff values were determined based on the maximum Youden index. For 11-KAD and 11-KT, the AUCs were not statistically significant (*P* > 0.05); therefore, optimal cutoff values were not calculated.

All 37 PCOS patients enrolled in this study had elevation of at least one conventional androgen (TT, FAI, AD, or DHEA-S). To evaluate whether combining TT and AD adds diagnostic value beyond FAI alone, we constructed a Venn diagram according to the method of Tosi et al. (19), using the 95th percentile cutoffs derived from the healthy control group (FAI >4.78, TT >1.72 nmol/L, AD >4.63 nmol/L). As shown in **Figure 2B**, FAI was elevated in 32 of 37 patients (86.5%). Among the five patients with normal FAI, four had elevated TT and/or AD: one with isolated TT elevation, one with isolated AD elevation, and two with elevation of both TT and AD. Consequently, the combination of FAI, TT, and AD identified 36 of 37 patients (97.3%). The single patient not captured by any of these three markers had isolated elevation of DHEA-S.

### 3.4 Changes in steroid hormones after the dexamethasone suppression test

**Figure 3:**
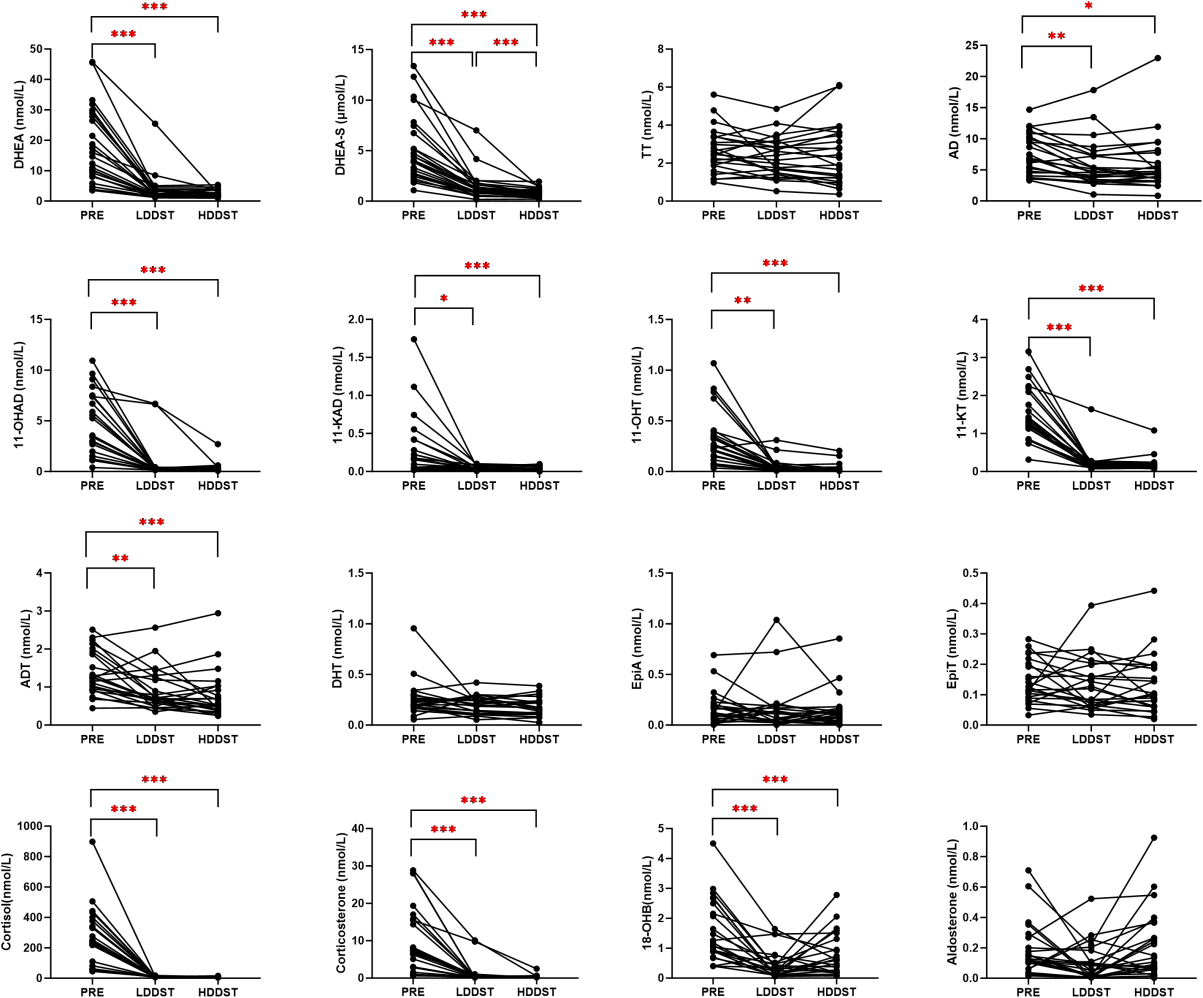
Individual trajectories of 12 androgens, cortisol, corticosterone, 18-hydroxycorticosterone, and aldosterone before and after dexamethasone suppression test in 24 PCOS patients. After dexamethasone administration, DHEA, DHEA-S, and 11-oxygenated androgens (11-OHAD, 11-KAD, 11-KT, and 11-OHT) were all significantly suppressed. Among the 24 PCOS patients, 4 patients (16.7%) and 7 patients (29.2%) showed significant suppression of testosterone and androstenedione (decrease below 50% of baseline values) after high-dose dexamethasone administration, respectively. Androsterone decreased significantly. Dihydrotestosterone showed an overall difference, but none of the pairwise comparisons reached statistical significance after Bonferroni correction. Epiandrosterone and epitestosterone showed no significant changes. Cortisol levels were suppressed to low levels. Corticosterone and 18−hydroxycorticosterone were also significantly suppressed after dexamethasone. Aldosterone did not decrease significantly after dexamethasone. PRE: pre−dexamethasone baseline; LDDST: low−dose dexamethasone suppression test; HDDST: high−dose dexamethasone suppression test. *P < 0.05, **P < 0.01, ***P < 0.001. All *P* values for pairwise comparisons in the figure are Bonferroni-corrected.

We did low-dose and high-dose dexamethasone suppression tests on 24 PCOS patients. **Supplementary Table 5** gives the full hormone data (glucocorticoids, mineralocorticoids, androgens, estrogens, progestogens). **Figure 3** shows the individual changes for 12 androgens, cortisol, corticosterone, 18-hydroxycorticosterone, and aldosterone. After suppression, cortisol levels (low−dose: 8.00 (6.35–9.25) nmol/L; high−dose: 8.06 (5.85–9.11) nmol/L) were well below the standard suppression threshold of 50 nmol/L (1.8 µg/dL) (20), confirming effective suppression of the hypothalamic-pituitary-adrenal axis. Corticosterone and 18−hydroxycorticosterone were also significantly suppressed after dexamethasone. In contrast, aldosterone, a mineralocorticoid not primarily regulated by ACTH, did not show a consistent decrease after dexamethasone.

DHEA, DHEA-S, and the 11-oxygenated androgens (11-OHAD, 11-KAD, 11-KT, and 11-OHT) all decreased significantly after dexamethasone suppression (all *P* < 0.05). After high-dose suppression, the median inhibition rates were 86.0%, 81.9%, 92.2%, 83.8%, 89.5%, and 89.3%, respectively. Androstenedione decreased significantly from baseline after both low-dose and high-dose suppression (*P* = 0.007 and *P* = 0.039), with a median inhibition rate of 35.9% after high-dose suppression. In contrast, testosterone showed no significant difference across the three time points (*P* = 0.522), with a median inhibition rate of 14.0%. Using a decrease of more than 50% from baseline after high-dose suppression as the criterion for significant suppression (applied only to testosterone and androstenedione), 4 patients (16.7%) showed significant suppression of testosterone and 7 patients (29.2%) showed significant suppression of androstenedione.

DHEA-S was used as an indicator of adrenal-derived androgen excess (21, 22). According to the 95th percentile reference value derived from the healthy control group (**Table 1**), 7 out of 24 patients (29.2%) had elevated baseline DHEA-S levels. However, elevated DHEA-S levels were not consistent with significant suppression of androgens after dexamethasone. Among the 4 patients with significant testosterone suppression, only 1 had elevated DHEA-S. Among the 7 patients with significant androstenedione suppression, only 4 had elevated DHEA-S. This finding suggests that most patients defined as having adrenal-origin dominance by the dexamethasone suppression test did not have elevated baseline DHEA-S levels.

Androsterone decreased significantly from baseline after high-dose suppression (*P* < 0.001), with a median inhibition rate of 54.8%. Dihydrotestosterone showed an overall statistically significant difference (*P* = 0.030), but after Bonferroni correction, none of the pairwise comparisons reached statistical significance (all *P* > 0.05). Epiandrosterone and epitestosterone showed no significant changes before or after dexamethasone suppression (*P* = 0.911 and *P* = 0.909, respectively).

## 4 Discussion

Hyperandrogenemia is a core biochemical feature of adolescent PCOS, and identifying its etiology is crucial for individualized treatment. PCOS has long been recognized as the most common cause of hyperandrogenemia in women of reproductive age (23–25), whereas non-classic adrenal hyperplasia (NCAH), classic congenital adrenal hyperplasia (CAH), and androgen-secreting tumors are much less frequent (26). Approximately 20–30% of PCOS patients show evidence of adrenal-derived androgen excess (27), suggesting that adrenal dysfunction contributes to PCOS pathogenesis. When assessing hyperandrogenemia, total testosterone and the free androgen index are first-line indicators recommended by international guidelines, but routine chemiluminescence immunoassays lack accuracy (28). LC-MS/MS is the gold standard for steroid detection and has been recommended for accurate androgen measurement (29). Therefore, we combined LC-MS/MS with a dexamethasone suppression test to comprehensively evaluate hyperandrogenemia in adolescent PCOS.

Using low-dose and high-dose dexamethasone suppression tests with LC-MS/MS, we systematically evaluated the suppressive response of 12 androgens for the first time in adolescent PCOS patients; a literature search confirmed no prior report on dexamethasone suppression of 11-oxygenated androgens. Previous studies suggested that a serum testosterone suppression >40% after low-dose dexamethasone can help distinguish ovarian from adrenal sources of androgen excess (30). In our study, all 11-oxygenated androgens showed a significant decrease after dexamethasone suppression (median inhibition rates > 80%), suggesting that their synthesis is regulated by ACTH and that their origin is likely primarily adrenal. As reviewed by Turcu and Auchus, although 11-oxygenated androgens are predominantly derived from the adrenal cortex, the 11-oxygenated testosterones (11-OHT and 11-KT) are formed peripherally from 11-OHAD (7, 31). In contrast, classic androgens (androstenedione, testosterone) showed heterogeneous responses; only 16.7% and 29.2% of patients had significant testosterone and androstenedione suppression (>50% decrease), respectively, suggesting that hyperandrogenemia in most PCOS patients originates from the ovaries. This is consistent with Chen et al., who reported similar suppression rates in adult PCOS (15).

DHEA-S, traditionally used as a marker of adrenal androgen excess, was elevated at baseline in 20.8% of patients (5/24). However, elevated DHEA-S levels did not parallel significant androgen suppression after dexamethasone: among patients with significant testosterone suppression (n=4) or androstenedione suppression (n=7), only 1 and 3 had elevated DHEA-S, respectively. Chen et al. similarly found that only 1 of 14 PCOS patients with elevated DHEA-S had significant testosterone suppression (15). DHEA-S likely reflects the overall secretory capacity of the adrenal zona reticularis (21, 22), whereas the dexamethasone suppression test assesses the efficiency of converting androgen precursors into active androgens; these two measures are not interchangeable. Notably, the scatter plot analysis further revealed that DHEA−S explained only a limited proportion of the variation in 11−OHAD, supporting the notion that circulating DHEA−S levels are largely determined by secretion-independent factors, which may be related to the heritability of DHEAS levels. As demonstrated by the Venn diagram analysis of Tosi et al. (19), isolated DHEAS elevation is a very nonspecific diagnostic indicator of PCOS, a view also reflected in international guidelines (2). Regarding epiandrosterone and epitestosterone, this is the first report of their lack of response to dexamethasone suppression in adolescent PCOS. Epitestosterone is mainly of gonadal origin, with minimal adrenal secretion (32). Epiandrosterone is a peripheral metabolite of DHEA produced via 5α-reductase and 3β-hydroxysteroid dehydrogenase in peripheral tissues (33). These hormones are primarily regulated by peripheral metabolic enzymes rather than ACTH and are not suitable markers of adrenal origin.

Comparison of low-dose versus high-dose dexamethasone (**Supplementary Table 5**) showed no significant differences for 11 of the 12 androgens, including all 11-oxygenated ones. In contrast, DHEA-S showed a further statistically significant decrease after high-dose dexamethasone (*P* < 0.001). This suggests that a low-dose regimen may be sufficient for most ACTH-dependent adrenal androgens, but DHEA-S requires higher or more prolonged suppression; as reported by Rosenfield et al. (34), time rather than dose can account for this effect due to slow DHEA-S metabolism.

To contextualize our findings with published data, we compiled all available LC-MS/MS studies (**Supplementary Table 4**). 11-KAD and 11-KT are derived from 11-OHAD via peripheral conversion through the following pathway: 11β-hydroxysteroid dehydrogenase type 2 (11β-HSD2) converts 11-OHAD to 11-KAD, which is then further converted to 11-KT by aldo-keto reductase 1C3 (AKR1C3, also known as 17β-hydroxysteroid dehydrogenase type 5) (31). Based on this metabolic order, one might theoretically expect 11-KAD levels to be higher than 11-KT. However, in our adolescent PCOS cohort, 11-KAD was lower relative to 11-OHAD, whereas 11-KT was higher relative to 11-KAD. A similar pattern was observed in healthy adolescent females in other studies (35, 36). The age-specific reference intervals established by Zeidler et al. (36) showed that 11-KT levels peak during adolescence and decline slightly thereafter. This dynamic change may explain why 11-KT appears relatively higher and 11-KAD relatively lower during adolescence, suggesting a possible link with the unique hormonal characteristics of this developmental stage. Nevertheless, findings across studies are not entirely consistent. Some studies in healthy adult women and Japanese women with PCOS (10, 36, 37) reported higher 11-KT relative to 11-KAD, whereas others (38, 39) observed lower 11-KT relative to 11-KAD in both healthy adults and adult PCOS patients. These discrepancies suggest that the 11−KAD to 11−KT ratio may be influenced by factors such as methodological differences, age, and geographic region, and its clinical significance warrants further investigation.

Our data also allowed us to compare adolescent and adult levels reported in the literature. In the present study, healthy adolescent controls had generally lower levels of 11-oxygenated C19 steroids than those reported in adults (38, 39). Zeidler et al. (36) demonstrated a progressive increase in four 11-oxygenated androgens from childhood through adulthood. A similar trend was observed for DHEA-S and DHEA, with adolescents (both healthy and PCOS) tending to have lower levels than their adult counterparts (15, 38). These results suggest that adrenarche may not be fully completed during adolescence. Nevertheless, given the limited number of studies on this topic, further validation is needed.

## 5 Limitations

This study has several limitations. First, the sample size was relatively small, which limited subgroup analyses and reduced the precision of the reference intervals (5th–95th percentiles) derived from the 22 healthy controls. Therefore, the reported percentiles should be interpreted as descriptive statistics of this cohort rather than established reference ranges, and large-scale validation studies are needed to determine robust reference intervals. Second, although we adjusted for BMI using bootstrap linear regression, residual confounding due to obesity cannot be completely ruled out. Third, for ethical and practical reasons, the dexamethasone suppression test did not include a healthy control group, although the risk of administering dexamethasone at these doses for 2–4 days is minimal; therefore, our suppression findings are exploratory. Fourth, this was a single-center study, and the results require validation in larger, multicenter cohorts. Fifth, our PCOS cohort consisted entirely of patients with biochemical hyperandrogenemia, which may limit the generalizability of our findings to PCOS patients without biochemical evidence of androgen excess (i.e., those with clinical hyperandrogenism only). Future studies should include larger samples, appropriate control groups where feasible, and basic experimental approaches to further elucidate the heterogeneous regulation of adrenal-derived androgens.

## 6 Conclusion

This study systematically evaluated the steroid hormone profile in adolescent PCOS patients using LC-MS/MS technology. PCOS patients show widespread androgen abnormalities, and FAI demonstrates the highest diagnostic value, with the addition of total testosterone and androstenedione modestly improving detection. The dexamethasone suppression test showed that DHEA, DHEA-S, and 11-oxygenated androgens were all significantly suppressed, supporting their adrenal origin, whereas testosterone suppression was minimal. Of note, elevated DHEA-S levels are not consistently aligned with adrenal-origin androgen dominance defined by the suppression test, indicating that using DHEA-S alone to assess the adrenal contribution to androgens may have limitations. However, due to the limited sample size, these findings warrant further validation in larger populations.

## Data Availability

The original contributions presented in the study are included in the article/**Supplementary Material**. Further inquiries can be directed to the corresponding author.
