# Supplementary material for: Androgen profiling in adolescent girls with polycystic ovary syndrome
Source: Front Endocrinol (Lausanne). 2026 Jun 3;17:1817505. doi: 10.3389/fendo.2026.1817505 (PMC13272079; doi:10.3389/fendo.2026.1817505)
Supplement: Supplementary file 1 [file Table1.docx]

**Abbreviations are the same as those defined in the main text.**

# Supplementary Table 1. Spearman correlation matrix of androgen parameters in the control group.

| **Variable** | **DHEA** | **DHEA-S** | **AD** | **TT** | **ADT** | **DHT** | **11-OHAD** | **11-KAD** | **11-KT** | **11-OHT** | **EpiA** | **EpiT** |
| --- | --- | --- | --- | --- | --- | --- | --- | --- | --- | --- | --- | --- |
| DHEA | 1.000 | 0.657** | 0.438* | 0.363 | 0.430* | 0.369 | 0.656** | 0.141 | 0.713** | 0.659** | -0.209 | 0.231 |
| DHEA-S | 0.657** | 1.000 | 0.152 | 0.091 | 0.287 | 0.038 | 0.500* | 0.036 | 0.444* | 0.483* | -0.189 | 0.028 |
| AD | 0.438* | 0.152 | 1.000 | 0.814** | 0.686** | 0.777** | 0.167 | 0.225 | 0.299 | 0.213 | -0.335 | 0.633** |
| TT | 0.363 | 0.091 | 0.814** | 1.000 | 0.799** | 0.844** | 0.109 | 0.013 | 0.347 | 0.276 | -0.191 | 0.698** |
| ADT | 0.430* | 0.287 | 0.686** | 0.799** | 1.000 | 0.870** | 0.150 | -0.056 | 0.365 | 0.421 | -0.414 | 0.712** |
| DHT | 0.369 | 0.038 | 0.777** | 0.844** | 0.870** | 1.000 | 0.155 | 0.097 | 0.433* | 0.349 | -0.293 | 0.668** |
| 11-OHAD | 0.656** | 0.500* | 0.167 | 0.109 | 0.150 | 0.155 | 1.000 | 0.477* | 0.864** | 0.719** | -0.282 | -0.290 |
| 11-KAD | 0.141 | 0.036 | 0.225 | 0.013 | -0.056 | 0.097 | 0.477* | 1.000 | 0.449* | 0.032 | -0.479* | -0.083 |
| 11-KT | 0.713** | 0.444* | 0.299 | 0.347 | 0.365 | 0.433* | 0.864** | 0.449* | 1.000 | 0.802** | -0.241 | -0.050 |
| 11-OHT | 0.659** | 0.483* | 0.213 | 0.276 | 0.421 | 0.349 | 0.719** | 0.032 | 0.802** | 1.000 | -0.206 | 0.022 |
| EpiA | -0.209 | -0.189 | -0.335 | -0.191 | -0.414 | -0.293 | -0.282 | -0.479* | -0.241 | -0.206 | 1.000 | -0.252 |
| EpiT | 0.231 | 0.028 | 0.633** | 0.698** | 0.712** | 0.668** | -0.290 | -0.083 | -0.050 | 0.022 | -0.252 | 1.000 |

Data are presented as Spearman correlation coefficients (rₛ). **P* < 0.05, ***P* < 0.01, ****P* < 0.001. N = 22.

# Supplementary Table 2. Between‑group comparisons of correlations (PCOS vs control group) for adrenal androgens (DHEA, DHEA‑S) with 11‑oxygenated androgens (11‑OHAD, 11‑KT).

| Variable | Y steroid | Control group (n=22) | PCOS group (n=37) | *P* value* |
| --- | --- | --- | --- | --- |
| DHEA‑S | 11‑OHAD | r = 0.500 | r = 0.629 | 0.253 |
| DHEA | 11‑OHAD | r = 0.656 | r = 0.782 | 0.178 |
| DHEA‑S | 11‑KT | r = 0.444 | r = 0.362 | 0.366 |
| DHEA | 11‑KT | r = 0.713 | r = 0.510 | 0.124 |

*Fisher’s Z transformation.

# Supplementary Table 3. Within‑group comparisons of correlations between adrenal androgens DHEA‑S and DHEA with the same 11‑oxygenated androgens (11‑OHAD, 11‑KT).

| Group (n) | Y steroid | r (DHEA‑S, Y) | r (DHEA, Y) | *P* value* |
| --- | --- | --- | --- | --- |
| PCOS (37) | 11‑OHAD | 0.629 | 0.782 | **0.022** |
| PCOS (37) | 11‑KT | 0.362 | 0.510 | 0.075 |
| Control (22) | 11‑OHAD | 0.500 | 0.656 | 0.144 |
| Control (22) | 11‑KT | 0.444 | 0.713 | **0.031** |

*Steiger’s Z test. *P*-values lower than 0.05 are marked in bold.

# Supplementary Table 4. Comparison of 11‑oxygenated androgens and DHEA‑S between the present study and published LC‑MS/MS data, stratified by age group.

| **Group [Ref.]** | **Country / Region** | **Age(year)** | **N** | **11‑OHAD (nmol/L)** | **11‑KAD (nmol/L)** | **11‑OHT (nmol/L)** | **11‑KT (nmol/L)** | **DHEA‑S (μmol/L)** | **DHEA (nmol/L)** |
| --- | --- | --- | --- | --- | --- | --- | --- | --- | --- |
| **Healthy adolescents** |  |  |  |  |  |  |  |  |  |
| Present study | China | 13 (12, 14) | 22 | 1.86 (1.27, 2.94) | 0.11 (0.05, 0.19) | 0.09 (0.05, 0.12) | 1.00 (0.83, 1.50) | 2.08 (1.06, 2.75) | 5.69 (4.65, 8.53) |
| Adriaansen et al. (1) ^b^ | Netherlands | 9-11 | 56 | 2.30 (1.66, 2.65) | 0.31 (0.26, 0.45) | 0.11 (0.08, 0.15) | 0.65 (0.49, 0.91) | — | 7.05 (5.10, 10.6) |
| Adriaansen et al. (1) ^b^ | Netherlands | 12-14 | 54 | 2.28 (1.51, 3.22) | 0.43 (0.30, 0.59) | 0.15 (0.11, 0.20) | 1.10 (0.72, 1.47) | — | 10.6 (7.35, 14.4) |
| Adriaansen et al. (1) ^b^ | Netherlands | 15-17 | 41 | 2.22 (1.75, 3.26) | 0.39 (0.27, 0.58) | 0.22 (0.15, 0.30) | 1.00 (0.64, 1.39) | — | 12.3 (7.83, 17.2) |
| Zeidler et al. (2) ^b^ | Germany | 10 - < 15 | 650 | 2.62 (0.93, 6.37) | 0.48 (0.17, 1.17) | 0.19 (<0.52) | 1.26 (0.51, 2.83) | — | — |
| Taylor et al. (3) | United States | 14 (13, 16) | 70 | 2.91 (2.05, 4.07) | — | 0.29 (0.21, 0.43) | 0.87 (0.57, 1.21) | 3.99 (2.04, 7.14) | — |
| Breslow et al. (4) ^a^ | United States | 11.7 ± 1.1 | 18 | 2.37 (0.94, 4.0) | — | 0.19 (0.06, 0.36) | 1.07 (0.40, 1.86) | — | — |
| Breslow et al. (4) ^a^ | United States | 14.1 ± 1.6 | 18 | 2.27 (1.82, 5.82) | — | 0.30 (0.09, 0.54) | 1.15 (0.69, 1.74) | — | — |
| Torchen et al. (5) ^c^ | United States | 10 ± 1 | 21 | — | — | — | — | 1.40 ± 1.06 | — |
| **Adolescent PCOS** |  |  |  |  |  |  |  |  |  |
| Present study | China | 13 (12, 15) | 37 | 3.58 (2.54, 6.57) | 0.17 (0.06, 0.38) | 0.25 (0.12, 0.34) | 1.30 (0.93, 1.63) | 4.25 (2.72, 6.25) | 13.31 (8.98, 22.78) |
| Taylor et al. (3) | United States | 16 (15, 17) | 115 | 3.87 (2.23, 5.92) | — | 0.42 (0.26, 0.56) | 0.84 (0.59, 1.41) | 5.32 (3.39, 8.44) | — |
| Torchen et al. (5) ^c^ | United States | 10 ± 1 | 17 | — | — | — | — | 1.70 ± 1.06 | — |
| **Healthy adults** |  |  |  |  |  |  |  |  |  |
| O'Reilly et al. (6) | United Kingdom | 28 (23, 32) | 49 | 6.8 (4.9, 12.5) | 2.7 (2.0, 3.9) | 0.2 (0.1, 0.3) | 1.5 (1.2, 1.8) | 6.0 (3.4, 9.6) | 7.1 (4.2, 11.8) |
| Swart et al. (7) ^c^ | United States | 23.6 ± 2.6 | 19 | 6.76 ± 5.98 | 1.82 ± 1.00 | 0.39 ± 0.20 | 0.75 ± 0.28 | — | 10.69 ± 10.14 |
| Mody et al. (8) | United States | 31 (25, 35.8) | 78 | 3.88 (2.82, 5.01) | 0.49 (0.41, 0.68) | 0.37 (0.23, 0.54) | 0.71 (0.54, 1.05) | — | — |
| Zeidler et al. (2) ^b^ | Germany | 20 - < 40 | 145 | 3.68 (1.16, 9.26) | 0.53 (0.18, 1.36) | 0.43 (0.11, 1.24) | 0.98 (0.35, 2.11) | — | — |
| Zeidler et al. (2) ^b^ | Germany | 40 - < 60 | 99 | 4.48 (2.22, 10.4) | 0.58 (0.23, 1.33) | 0.60 (0.23, 1.32) | 1.15 (0.41, 2.47) | — | — |
| Chen et al. (9) ^c^ | China | 29.39 ± 4.23 | 41 | — | — | — | — | 6.35 ± 2.58 | 34.85 ± 17.68 |
| **Adult PCOS** |  |  |  |  |  |  |  |  |  |
| O'Reilly et al. (6) | United Kingdom | 30 (24, 36) | 114 | 31.7 (16.8, 47.8) | 13.4 (8.5, 18.8) | 0.4 (0.3, 0.5) | 2.4 (1.8, 3.9) | 8.1 (5.5, 12.2) | 14.1 (10.4, 18.2) |
| Swart et al. (7) ^c^ | United States | 20.4 ± 3.9 | 35 | 8.82 ± 5.99 | 1.30 ± 0.98 | 0.37 ± 0.24 | 1.08 ± 0.56 | — | 26.09 ± 15.93 |
| Mody et al. (8) | United States | 27 (24, 32) | 114 | 7.93 (5.14, 11.46) | 1.57 (0.88, 2.83) | 0.57 (0.42, 0.72) | 1.18 (0.76, 1.59) | — | — |
| Yoshida et al. (10) | Japan | 29.5 | 28 | 4.63 (3.33, 5.82) | 1.29 (1.07, 1.87) | 0.54 (0.42, 0.83) | 2.14 (1.78, 3.66) | — | — |
| Chen et al. (9) ^c^ | China | 29.02 ± 3.75 | 102 | — | — | — | — | 8.00 ± 2.87 | 36.47 ± 22.26 |

For the hormones 11‑OHAD, 11‑KAD, 11‑OHT, 11‑KT, DHEA‑S, and DHEA, data are presented as median (P25, P75) unless otherwise indicated by superscript symbols (a, b, c) attached to the group name (a: P10–P90; b: P2.5–P97.5; c: mean ± SD). —, not measured or not reported. For the Germany cohort (2), the 11‑OHT values below the lower limit of quantification are reported as <0.52 (P97.5). To ensure comparability across studies, data from selected references (Taylor et al. (3), Torchen et al. (5), Mody et al. (8), Yoshida et al. (10), and Chen et al. (9)) were converted to the units (nmol/L or μmol/L) used in the present study. Conversion factors from SI units to conventional units for the steroids listed in this table are the same as those provided in Table 1 (see parentheses in the Parameter column of Table 1).

**References**

1. Adriaansen BPH, Oude Alink SE, Swinkels DW, Schröder MAM, Span PN, Sweep FCGJ, Claahsen-van Der Grinten HL, Van Herwaarden AE. Reference intervals for serum 11-oxygenated androgens in children. *European Journal of Endocrinology* (2024) 190:96–103. doi: 10.1093/ejendo/lvae008

2. Zeidler R, Wagner F, Ceglarek U, Kiess W, Kratzsch J, Baber R, Wirkner K, Isermann B, Gaudl A, Vogel M, et al. Age- and sex-specific reference intervals for 11-oxygenated androgens from infancy throughout childhood and adulthood. *Clin Chem Lab Med* (2026) doi: 10.1515/cclm-2025-1440

3. Taylor AE, Ware MA, Breslow E, Pyle L, Severn C, Nadeau KJ, Chan CL, Kelsey MM, Cree-Green M. 11-Oxyandrogens in Adolescents With Polycystic Ovary Syndrome. *J Endocr Soc* (2022) 6:bvac037. doi: 10.1210/jendso/bvac037

4. Breslow E, Taylor A, Chan CL, Severn C, Pyle L, Torchen L, Sisk R, Legro R, Turcu AF, Auchus RJ, et al. 11-Oxygenated Androgen Metabolite Concentrations Are Affected by Pubertal Progression and Obesity. *Horm Res Paediatr* (2023) 96:412–422. doi: 10.1159/000528341

5. Torchen LC, Sisk R, Legro RS, Turcu AF, Auchus RJ, Dunaif A. 11-Oxygenated C19 Steroids Do Not Distinguish the Hyperandrogenic Phenotype of PCOS Daughters from Girls with Obesity. *The Journal of Clinical Endocrinology & Metabolism* (2020) 105:e3903–e3909. doi: 10.1210/clinem/dgaa532

6. O’Reilly MW, Kempegowda P, Jenkinson C, Taylor AE, Quanson JL, Storbeck K-H, Arlt W. 11-Oxygenated C19 Steroids Are the Predominant Androgens in Polycystic Ovary Syndrome. *The Journal of Clinical Endocrinology & Metabolism* (2017) 102:840–848. doi: 10.1210/jc.2016-3285

7. Swart AC, du Toit T, Gourgari E, Kidd M, Keil M, Faucz FR, Stratakis CA. Steroid hormone analysis of adolescents and young women with polycystic ovarian syndrome and adrenocortical dysfunction using UPC2 -MS/MS. *Pediatr Res* (2021) 89:118–126. doi: 10.1038/s41390-020-0870-1

8. Mody AP, Lodish MB, Auchus RJ, Turcu AF, Jiang F, Huddleston HG. Exploring the Predictive Role of 11‐Oxyandrogens in Diagnosing Polycystic Ovary Syndrome. *Endocrino Diabet &amp; Metabol* (2025) 8:e70022. doi: 10.1002/edm2.70022

9. Chen F, Chen M, Zhang W, Yin H, Chen G, Huang Q, Yang X, Chen L, Lin C, Yin G. Comparison of the efficacy of different androgens measured by LC-MS/MS in representing hyperandrogenemia and an evaluation of adrenal-origin androgens with a dexamethasone suppression test in patients with PCOS. *J Ovarian Res* (2021) 14:32. doi: 10.1186/s13048-021-00781-5

10. Yoshida T, Matsuzaki T, Miyado M, Saito K, Iwasa T, Matsubara Y, Ogata T, Irahara M, Fukami M. 11-oxygenated C19 steroids as circulating androgens in women with polycystic ovary syndrome. *Endocr J* (2018) 65:979–990. doi: 10.1507/endocrj.EJ18-0212

# Supplementary Table 5. Steroid hormone profiles at baseline and following low- and high-dose dexamethasone suppression tests in girls with hyperandrogenemia (n=24).

| **Variables** | **PRE** | **LDDST** | **HDDST** | ***P* value** | **PRE vs LDDST** | **PRE vs HDDST** | **LDDST vs HDDST** |
| --- | --- | --- | --- | --- | --- | --- | --- |
| **Androgens** |  |  |  |  |  |  |  |
| DHEA (nmol/L) | 15.32 (8.53, 28.67) | 2.29 (1.80, 3.78) | 2.15 (1.56, 3.54) | **<0.001** | **<0.001** | **<0.001** | 0.144 |
| DHEA-S (μmol/L) | 4.23 (2.69, 7.27) | 1.21 (0.79, 1.74) | 0.87±0.43 | **<0.001** | **<0.001** | **<0.001** | **<0.001** |
| AD (nmol/L) | 7.39±3.23 | 4.72 (3.43, 7.34) | 4.40 (3.62, 7.35) | **0.011** | **0.007** | **0.039** | 1.000 |
| TT (nmol/L) | 2.44±0.98 | 2.31±0.86 | 2.28±1.39 | 0.522 | — | — | — |
| ADT (nmol/L) | 1.41±0.56 | 0.71 (0.56, 1.27) | 0.57 (0.43, 1.03) | **0.003** | **0.006** | **<0.001** | 1.000 |
| DHT (nmol/L) | 0.20 (0.17, 0.29) | 0.20±0.09 | 0.18±0.09 | **0.030** | 0.707 | 0.086 | 0.321 |
| 11-OHAD (nmol/L) | 4.72±3.05 | 0.27 (0.19, 0.34) | 0.28 (0.16, 0.42) | **<0.001** | **<0.001** | **<0.001** | 1.000 |
| 11-KAD (nmol/L) | 0.16 (0.04, 0.42) | 0.04 (0.02, 0.06) | 0.03 (0.01, 0.04) | **<0.001** | **0.018** | **<0.001** | 0.179 |
| 11-KT (nmol/L) | 1.40±0.71 | 0.16 (0.12, 0.23) | 0.13 (0.09, 0.20) | **<0.001** | **<0.001** | **<0.001** | 0.138 |
| 11-OHT (nmol/L) | 0.23 (0.11, 0.39) | 0.03 (0.01, 0.04) | 0.02 (0.01, 0.04) | **<0.001** | **0.001** | **<0.001** | 1.000 |
| EpiA (nmol/L) | 0.12 (0.05, 0.21) | 0.11 (0.03, 0.17) | 0.10 (0.04, 0.15) | 0.727 | — | — | — |
| EpiT (nmol/L) | 0.14±0.07 | 0.12 (0.07, 0.20) | 0.10 (0.06, 0.19) | 0.321 | — | — | — |
| **Glucocorticoids** |  |  |  |  |  |  |  |
| 11DOF (nmol/L) | 1.24 (0.57, 1.96) | 0.07 (0.05, 0.09) | 0.07 (0.04, 0.09) | **<0.001** | **<0.001** | **<0.001** | 1.000 |
| F (nmol/L) | 242.09 (215.66, 367.35) | 8.00 (6.35, 9.24) | 8.06 (5.85, 9.10) | **<0.001** | **<0.001** | **<0.001** | 1.000 |
| E (nmol/L) | 50.94±17.40 | 2.50 (1.94, 3.33) | 2.36 (1.66, 2.75) | **<0.001** | **<0.001** | **<0.001** | **0.035** |
| 21DOF (nmol/L) | 0.05 (0.01, 0.08) | 0.01 (0.00, 0.01) | 0.01 (0.01, 0.01) | **<0.001** | **0.001** | **0.001** | 1.000 |
| 18-OHF (nmol/L) | 1.52±0.81 | 0.08 (0.07, 0.14) | 0.11 (0.07, 0.17) | **<0.001** | **<0.001** | **<0.001** | 1.000 |
| 18-OF (nmol/L) | 0.05 (0.02, 0.06) | 0.00 (0.00, 0.01) | 0.01 (0.00, 0.02) | **<0.001** | **<0.001** | **<0.001** | 0.294 |
| **Mineralocorticoids** |  |  |  |  |  |  |  |
| 11-DOC (nmol/L) | 0.09 (0.05, 0.15) | 0.02 (0.02, 0.05) | 0.02 (0.02, 0.04) | **<0.001** | **<0.001** | **<0.001** | 1.000 |
| B (nmol/L) | 7.85 (3.52, 15.79) | 0.20 (0.12, 0.35) | 0.32 (0.17, 0.49) | **<0.001** | **<0.001** | **<0.001** | 0.380 |
| 18-OHB (nmol/L) | 1.18 (0.88, 2.50) | 0.25 (0.10, 0.57) | 0.59 (0.21, 1.31) | **<0.001** | **<0.001** | **<0.001** | 0.173 |
| ALD (nmol/L) | 0.13 (0.07, 0.25) | 0.05 (0.01, 0.18) | 0.12 (0.05, 0.34) | **0.009** | 0.053 | 1.000 | **0.017** |
| **Estrogens** |  |  |  |  |  |  |  |
| E1 (pmol/L) | 138.33 (108.63, 193.37) | 137.07±44.35 | 107.41 (90.25, 162.56) | **0.032** | 0.068 | **0.021** | 1.000 |
| E2 (pmol/L) | 124.82 (86.94, 171.89) | 124.46 (70.12, 176.22) | 129.38±67.88 | 0.878 | — | — | — |
| E3 (pmol/L) | 2.53 (1.35, 7.66) | 2.77±2.32 | 2.18 (1.07, 4.89) | 0.302 | — | — | — |
| **Progestogens** |  |  |  |  |  |  |  |
| PREG (nmol/L) | 1.16±0.82 | 0.29 (0.15, 0.66) | 0.20 (0.10, 0.37) | **0.001** | **0.005** | **0.001** | 0.744 |
| P (nmol/L) | 0.25 (0.16, 0.41) | 0.16 (0.10, 0.25) | 0.13 (0.10, 0.25) | **0.001** | 0.192 | 0.059 | 1.000 |
| 17-OHPREG (nmol/L) | 5.02 (3.64, 10.20) | 0.45 (0.33, 0.90) | 0.51 (0.39, 0.87) | **<0.001** | **<0.001** | **<0.001** | 1.000 |
| 17-OHP (nmol/L) | 2.06 (1.45, 3.15) | 1.36 (1.15, 2.30) | 1.76±0.91 | **0.019** | 0.067 | 0.093 | 1.000 |

Normally distributed data are presented as mean ± SD; non-normally distributed data are presented as median (25th percentile, 75th percentile). For variables with normal distribution at all three time points, repeated-measures ANOVA was used for overall comparison. If Mauchly's test of sphericity was significant (*P* < 0.05), the Greenhouse-Geisser correction was applied. Post-hoc pairwise comparisons were performed using the paired t-test with Bonferroni correction. For variables with non-normal distribution at any time point, the Friedman test was used for overall comparison, and post-hoc pairwise comparisons were performed using the Wilcoxon signed-rank test with Bonferroni correction. All pairwise *P* values presented are Bonferroni-corrected. PRE, pre-dexamethasone; LDDST, low-dose dexamethasone suppression test; HDDST, high-dose dexamethasone suppression test. *P*-values lower than 0.05 are marked in bold.

# Supplementary Table 6. Method performance characteristics of steroid hormones.

| Analyte | Intra-assay CV(%) |  |  | Inter-assay CV (%) |  |  | Recovery(%) |  |  | Linear range | Unit | LOQ | LOD |
| --- | --- | --- | --- | --- | --- | --- | --- | --- | --- | --- | --- | --- | --- |
|  | Low | Med | High | Low | Med | High | Low | Med | High |  |  |  |  |
| PREG | 10.6 | 5.2 | 4.3 | 10.3 | 10.0 | 12.2 | 96 | 98 | 91 | 125-50000 | pg/mL | 125 | 31 |
| P | 9.9 | 3.7 | 3.5 | 7.3 | 7.0 | 9.8 | 94 | 96 | 93 | 0.125-50 | ng/mL | 0.0125 | 0.00625 |
| 11-DOC | 3.5 | 1.5 | 3.1 | 3.2 | 3.9 | 3.3 | 100 | 99 | 95 | 25-10000 | pg/mL | 2.5 | 1.25 |
| B | 6.2 | 2.1 | 3.7 | 7.3 | 8.1 | 9.5 | 97 | 95 | 97 | 0.05-20 | ng/mL | 0.005 | 0.0025 |
| 18-OHB | 1.6 | 2.1 | 1.8 | 3.6 | 2.2 | 3.3 | 95 | 107 | 102 | 50-20000 | pg/mL | 25 | 12.5 |
| ALD | 10.8 | 4.2 | 4.1 | 11.5 | 4.8 | 3.0 | 93 | 97 | 103 | 12.5-5000 | pg/mL | 12.5 | 5 |
| 17-OHPREG | 4.7 | 2.2 | 2.9 | 3.4 | 3.3 | 3.4 | 97 | 98 | 97 | 0.1-40 | ng/mL | 0.1 | 0.025 |
| 17-OHP | 6.4 | 1.9 | 2.1 | 4.8 | 4.2 | 4.2 | 109 | 101 | 97 | 0.05-20 | ng/mL | 0.005 | 0.0025 |
| 11-DOF | 2.7 | 2.1 | 2.3 | 3.2 | 2.4 | 2.3 | 105 | 100 | 100 | 50-20000 | pg/mL | 25 | 12.5 |
| 21-DOF | 3.3 | 1.5 | 3.6 | 4.4 | 2.6 | 2.5 | 103 | 97 | 100 | 25-10000 | pg/mL | 25 | 12.5 |
| F | 2.1 | 1.5 | 3.6 | 4.3 | 3.0 | 4.6 | 104 | 100 | 101 | 0.75-300 | ng/mL | 0.12 | 0.075 |
| 18-OHF | 3.0 | 1.7 | 1.9 | 4.2 | 2.1 | 3.2 | 99 | 99 | 104 | 25-10000 | pg/mL | 6.25 | 2.5 |
| 18-OF | 4.5 | 1.2 | 1.9 | 6.3 | 3.7 | 3.7 | 96 | 93 | 102 | 10-2000 | pg/mL | 10 | 5 |
| E | 2.1 | 1.3 | 3.5 | 2.1 | 2.3 | 2.9 | 105 | 99 | 100 | 0.125-50 | ng/mL | 0.0125 | 0.00625 |
| DHEA | 7.6 | 3.4 | 4.0 | 10.1 | 5.1 | 6.5 | 96 | 88 | 95 | 0.125-50 | ng/mL | 0.125 | 0.03 |
| DHEA-S | 0.8 | 2.1 | 2.4 | 4.7 | 3.1 | 3.1 | 101 | 98 | 98 | 12.5-5000 | ng/mL | 2.5 | 1.25 |
| AD | 1.7 | 2.0 | 2.0 | 2.3 | 1.8 | 2.3 | 102 | 101 | 100 | 50-20000 | pg/mL | 7.5 | 5 |
| 11-OHAD | 2.6 | 1.2 | 2.8 | 3.2 | 3.6 | 3.1 | 102 | 101 | 104 | 50-20000 | pg/mL | 50 | 25 |
| 11-KAD | 5.0 | 5.1 | 3.2 | 6.7 | 10.6 | 5.1 | 91 | 89 | 101 | 12.5-2500 | pg/mL | 25 | 6.25 |
| E1 | 2.5 | 3.8 | 2.9 | 9.9 | 6.0 | 5.8 | 81 | 90 | 95 | 5-2000 | pg/mL | 5 | 1 |
| TT | 2.4 | 1.5 | 2.6 | 3.3 | 2.7 | 2.0 | 102 | 100 | 100 | 50-20000 | pg/mL | 7.5 | 5 |
| 11-OHT | 4.1 | 2.6 | 2.5 | 5.8 | 4.9 | 3.1 | 109 | 102 | 105 | 12.5-5000 | pg/mL | 6.25 | 3 |
| 11-KT | 2.1 | 1.3 | 3.6 | 3.8 | 3.4 | 2.6 | 111 | 104 | 107 | 25-10000 | pg/mL | 5 | 2.5 |
| E2 | 8.7 | 5.6 | 3.4 | 15.5 | 14.7 | 5.7 | 77 | 91 | 101 | 5-2000 | pg/mL | 5 | 1 |
| E3 | 16.3 | 6.7 | 3.0 | 15.5 | 6.6 | 4.5 | 89 | 93 | 106 | 5-2000 | pg/mL | 5 | 1 |
| DHT | 8.0 | 1.6 | 2.0 | 6.6 | 4.8 | 4.0 | 98 | 97 | 96 | 25-10000 | pg/mL | 25 | 6.25 |
| EpiT | 5.7 | 2.3 | 3.8 | 4.9 | 4.8 | 4.8 | 118 | 115 | 110 | 25-10000 | pg/mL | 5 | 2.5 |
| ADT | 13.4 | 8.8 | 4.6 | 14.3 | 9.4 | 9.6 | 96 | 92 | 85 | 100-10000 | pg/mL | 100 | 50 |
| EpiA | 4.2 | 7.4 | 3.5 | 10.3 | 7.6 | 5.1 | 91 | 103 | 99 | 250-10000 | pg/mL | 250 | 100 |

CV: coefficient of variation; LOD: limit of detection; LOQ: limit of quantification; Med: medium.

# Supplementary Table 7. Gradient elution program for LC-MS/MS.

| **Step** | **Time (min)** | **Flow rate (mL/min)** | **Aqueous phase (%)** | **Organic phase (%)** | **Curve** |
| --- | --- | --- | --- | --- | --- |
| 1 | Initial | 0.3 | 60 | 40 | Initial |
| 2 | 0.50 | 0.3 | 60 | 40 | 6 |
| 3 | 4.00 | 0.3 | 45 | 55 | 6 |
| 4 | 7.50 | 0.3 | 25 | 75 | 6 |
| 5 | 8.20 | 0.3 | 10 | 90 | 6 |
| 6 | 8.30 | 0.5 | 2 | 98 | 6 |
| 7 | 9.00 | 0.5 | 2 | 98 | 6 |
| 8 | 10.00 | 0.3 | 60 | 40 | 1 |

Aqueous phase: 0.5 mM ammonium fluoride in water; Organic phase: methanol.

# Supplementary Table 8. Mass spectrometric parameters for steroid hormones.

| **Analyte** | **Ionization mode** | **Quantifier transition (m/z)** | **Collision energy (eV)** | **Cone voltage (V)** | **Retention time (min)** |
| --- | --- | --- | --- | --- | --- |
| PREG | ESI+ | 299.10 > 281.06 | 14 | 30 | 7.44 |
| P | ESI+ | 315.30 > 109.02 | 10 | 50 | 7.09 |
| 11-DOC | ESI+ | 331.30 > 109.06 | 15 | 40 | 5.74 |
| B | ESI+ | 347.30 > 121.08 | 18 | 50 | 4.68 |
| 18-OHB | ESI+ | 363.2 > 269.1 | 16 | 15 | 3.35 |
| ALD | ESI- | 359.23 > 189.11 | 18 | 20 | 2.97 |
| 17-OHPREG | ESI- | 331.08 > 287.16 | 20 | 50 | 6.14 |
| 17-OHP | ESI+ | 331.30 > 97.04 | 15 | 40 | 6.18 |
| 11-DOF | ESI+ | 347.30 > 109.05 | 16 | 50 | 4.86 |
| 21-DOF | ESI+ | 347.27 > 311.23 | 14 | 50 | 4.45 |
| F | ESI+ | 363.30 > 327.19 | 7 | 50 | 3.77 |
| 18-OHF | ESI+ | 379.2 > 267.2 | 28 | 18 | 2.61 |
| 18-OF | ESI+ | 377.2 > 313.2 | 40 | 20 | 2.28 |
| E | ESI+ | 361.23 > 121.04 | 32 | 50 | 3.4 |
| DHEA | ESI+ | 271.23 > 213.20 | 15 | 40 | 6.01 |
| AD | ESI+ | 287.30 > 109.06 | 13 | 50 | 5.43 |
| 11-OHAD | ESI+ | 303.2 > 145.2 | 40 | 26 | 4.18 |
| 11-KAD | ESI+ | 301.2 > 121.1 | 30 | 20 | 3.57 |
| TT | ESI+ | 289.30 > 109.08 | 12 | 50 | 5.91 |
| 11-OHT | ESI+ | 305.2 > 269.2 | 14 | 14 | 4.58 |
| 11-KT | ESI+ | 303.2 > 121.1 | 40 | 22 | 3.98 |
| DHT | ESI+ | 291.30 > 255.28 | 16 | 50 | 6.66 |
| E1 | ESI- | 269.20 > 145.04 | 38 | 40 | 5.26 |
| E2 | ESI- | 271.04 > 183.06 | 40 | 30 | 5.33 |
| E3 | ESI- | 287.04 > 171.06 | 35 | 50 | 2.58 |
| EpiT | ESI+ | 289.3 > 97.1 | 40 | 20 | 6.79 |
| ADT | ESI+ | 273.3 > 147.2 | 30 | 20 | 7.54 |
| EpiA | ESI+ | 273.3 > 147.2 | 30 | 20 | 6.43 |
| DHEA-S | ESI- | 367.17 > 80.00 | 62 | 50 | 3.54 |

# Supplementary Table 9. Chemicals, reagents, standards and internal standards.

| **Category** | **No.** | **Name** | **Manufacturer** | **Catalog No.** |
| --- | --- | --- | --- | --- |
| **Reagents** | 1 | Methanol (HPLC grade, 4L) | Thermo Fisher | A452-4 |
|  | 2 | Acetonitrile (HPLC grade, 4L) | Thermo Fisher | A998-4 |
|  | 3 | Water (distilled) | Watsons | NA |
|  | 4 | Ammonium fluoride (25 g/bottle) | Sigma-Aldrich | 338869 |
| **Standards** | 1 | PREG | Cambridge | ULM-9159-0.01 |
|  | 2 | P | Sigma | V900699 |
|  | 3 | 11-DOC | IsoSciences | 13406 |
|  | 4 | B | TRC | C695700 |
|  | 5 | 18-OHB | Cerilliant | H-106 |
|  | 6 | ALD | Cerilliant | A-096 |
|  | 7 | 17-OHPREG | Cambridge | ULM-9155-D |
|  | 8 | 17-OHP | TRC | H952330 |
|  | 9 | 11-DOF | IsoSciences | 9012UNL |
|  | 10 | 21-DOF | Cambridge | ULM-9987-0.001 |
|  | 11 | F | TRC | TRC |
|  | 12 | 18-OHF | CMSS | CMS-T3100360 |
|  | 13 | 18-OF | TRC | O870615 |
|  | 14 | E | Dr Ehrenstorfer | C11705400 |
|  | 15 | DHEA | Cambridge | ULM-9143-0.01 |
|  | 16 | DHEA-S | Medical Isotopes | 30930 |
|  | 17 | AD | Dr Ehrenstorfer | C10255030 |
|  | 18 | 11-OHAD | Steraloids Inc | A6630 |
|  | 19 | 11-KAD | Steraloids Inc | A7250 |
|  | 20 | TT | Dr Ehrenstorfer | C17322500 |
|  | 21 | 11-OHT | Steraloids Inc | A5760 |
|  | 22 | 11-KT | Steraloids Inc | A6720 |
|  | 23 | DHT | Cerilliant | D-073 |
|  | 24 | E1 | TRC | E889050 |
|  | 25 | E2 | Dr Ehrenstorfer | C13213100 |
|  | 26 | E3 | Dr Ehrenstorfer | C13213200 |
|  | 27 | EpiT | A ChemTek Inc. | 1ST2266 |
|  | 28 | ADT | A ChemTek Inc. | 1ST8508 |
|  | 29 | EpiA | A ChemTek Inc. | 1ST2201-100A |
| **Internal standards** | 1 | PREG-D4 | Cambridge | DLM-6896-0.01 |
|  | 2 | P-D9 | Cerilliant | P-070 |
|  | 3 | 11-DOC-D8 | Medical Isotopes | D4301 |
|  | 4 | 18-OHB-D4 | IsoSciences | S8066-0.1 |
|  | 5 | ALD-D7 | IsoSciences | 5093 |
|  | 6 | 17-OHPREG-D2 C2 | Cambridge | CDLM-9154-0.001 |
|  | 7 | 17-OHP-D8 | CNW | D-5650 |
|  | 8 | 11-DOF-D5 | IsoSciences | 9012 |
|  | 9 | 21-DOF-D8 | Cambridge | DLM-8305-0.01 |
|  | 10 | F-D4 | TRC | C696302 |
|  | 11 | 18-OHF-D4 | Cambridge | DLM-10006-C |
|  | 12 | E-D8 | TRC | C696502 |
|  | 13 | DHEA-D6 | IsoSciences | 5170 |
|  | 14 | DHEA-S-D6 | Medical Isotopes | D17974 |
|  | 15 | AD-C3 | Sigma | 730645 |
|  | 16 | 11-OHAD-D4 | IsoSciences | 16148 |
|  | 17 | TT-C3 | Cerilliant | T-070 |
|  | 18 | 11-KT-D3 | IsoSciences | 16144 |
|  | 19 | DHT-C3 | Sigma | 730637 |
|  | 20 | E1-D4 | Medical Isotopes | D171 |
|  | 21 | E2-D4 | Cambridge | DLM-2487-5 |
|  | 22 | E3-D3 | Cambridge | DLM-8586-0.005 |
|  | 23 | ADT-D4 | CMASS | 361432-60-2 |

Dr. Ehrenstorfer (Dr. Ehrenstorfer GmbH, Germany); Cerilliant (Cerilliant Corporation, USA); TRC (Toronto Research Chemicals, Canada); IsoSciences (IsoSciences, USA); Cambridge (Cambridge Isotope Laboratories, USA); Medical Isotopes (Medical Isotopes, USA); Steraloids Inc (Steraloids Inc., USA); A ChemTek Inc. (A ChemTek Inc., USA); CMASS (CMASS Chemical Reagents, China); CNW (CNW Technologies, Germany); Sigma/Sigma-Aldrich (Merck KGaA, Germany); Thermo Fisher (Thermo Fisher Scientific, USA); Watsons (Watsons Water, China).
